# Cognitive and Behavioral Predictors of Light Therapy Use

**DOI:** 10.1371/journal.pone.0039275

**Published:** 2012-06-13

**Authors:** Kathryn A. Roecklein, Julie A. Schumacher, Megan A. Miller, Natalie C. Ernecoff

**Affiliations:** 1 Department of Psychology, University of Pittsburgh, Pittsburgh, Pennsylvania, United States of America; 2 Department of Psychiatry and Human Behavior, University of Mississippi Medical Center, Jackson, Mississippi, United States of America; University of Granada, Spain

## Abstract

**Objective:**

Although light therapy is effective in the treatment of seasonal affective disorder (SAD) and other mood disorders, only 53–79% of individuals with SAD meet remission criteria after light therapy. Perhaps more importantly, only 12–41% of individuals with SAD continue to use the treatment even after a previous winter of successful treatment.

**Method:**

Participants completed surveys regarding (1) social, cognitive, and behavioral variables used to evaluate treatment adherence for other health-related issues, expectations and credibility of light therapy, (2) a depression symptoms scale, and (3) self-reported light therapy use.

**Results:**

Individuals age 18 or older responded (*n* = 40), all reporting having been diagnosed with a mood disorder for which light therapy is indicated. Social support and self-efficacy scores were predictive of light therapy use (*p*'s<.05).

**Conclusion:**

The findings suggest that testing social support and self-efficacy in a diagnosed patient population may identify factors related to the decision to use light therapy. Treatments that impact social support and self-efficacy may improve treatment response to light therapy in SAD.

## Introduction

Although light therapy is effective in the treatment of seasonal affective disorder (SAD) [1], or Major Depressive Disorder with a Seasonal Pattern, as well as for non-seasonal depression [2], data suggest that only about 41% (24 out of 59) of SAD patients treated successfully with light therapy (LT) report regularly using the treatment in follow-up studies [3]. In another study combining LT and cognitive behavioral therapy (CBT), only 12% (4 out of 34) of individuals with SAD originally treated with LT alone (*n* = 19) or LT plus CBT (*n* = 15) used LT during the following winter [4]. These data suggest that the choice to use light therapy is itself a target for intervention. In the present study, we aimed to test whether certain motivational and social cognitive processes may help explain the decision to utilize LT.

Two separate issues exist, 1) LT use during the acute treatment phase, and 2) LT use in subsequent winters. However, similar factors may be associated with LT use in both instances. Among those using LT, only 53% of all those with SAD and 43% of moderate to severe cases meet remission criteria after LT [5]. A more recent light therapy trial found higher remission rates, ranging from 46% to 79%, depending on the stringency of remission criteria [6]. These remission rates compare favorably to remission rates for antidepressant medications, which ranged from 43% to 62% in recent meta-analyses and pooled analyses [7]–[9]. Maximizing remission rates may require higher rates of adherence to LT prescriptions. Adherence to LT in SAD is lower than hoped, about 41%–60% [3], [10]–[11] when treatment dropouts are included, and this incomplete adherence may explain the incomplete remission rates. The general literature on medication and medical treatment compliance suggests dose-taking compliance declines as the number of daily doses increases [12], and depression symptoms are associated with noncompliance [13]. Light therapy requires approximately 30–45 minutes a day, which represents a significant time commitment. Factors associated with light therapy use and adherence could be clinically relevant if such factors could be manipulated to help improve treatment outcomes.

In the one study to date assessing possible explanations for not using light therapy, 59 patients originally treated with light therapy were reassessed approximately 9 years later [3]. Among the 56% (*n* = 33 out of 59) who had discontinued using light therapy, 14% (n = 8) reported they had not had sufficient symptoms, leaving 44% of individuals who did have sufficient symptoms to warrant treatment but chose not to use light therapy. Those individuals reported inconvenience and/or perceived ineffectiveness as reasons for not using light therapy despite having experienced improvement during initial light therapy treatment. To examine further which factors predict light therapy use, we reviewed research on treatment adherence in depression and other similar health conditions in which treatment requires a time commitment.

The Transtheoretical Model [14]–[15], and Social Cognitive Theory [16] both describe variables that have been identified as important predictors of compliance with treatments such as continuous positive airway pressure (CPAP) treatment for obstructive sleep apnea, a treatment requiring nightly use of a CPAP mask and machine somewhat similar to the daily use required for light therapy [17]. Factors such as self-efficacy and depression symptoms have also been associated with adherence to treatments for depression. The Theory of Planned Behavior hypothesizes that tangible barriers (e.g., access to care), illness barriers (e.g., depression symptoms, concerns about treatment), and psychological barriers (e.g., self-efficacy, stigma) are modifiable and, therefore, targets of interventions to improve adherence to medication treatment of depression [18]. An intervention based on this model that incorporates motivational interviewing, problem solving, and psychoeducation has shown a significant increase in adherence to medications in older adults with depression from baseline across 24 weeks compared to treatment as usual [18].

Although different in their mechanisms of efficacy, homework within the context of psychotherapy has similar duration and daily frequency demands as light therapy. A recent meta-analysis of 23 studies on homework found that homework compliance had a small to medium effect (*r* = .26) on treatment outcome [19]. Individuals with more severe or longer-lasting symptoms comply less with homework [20]. Variables including motivation, readiness to change, and necessary skills are correlated with homework compliance and treatment outcome in cognitive behavioral therapy for depression [21]–[22].

The above data on factors associated with adherence to either homework in psychotherapy or medication treatment for depression utilize constructs from multiple theories of behavior change. Fishbein et al. (2001) [23] proposed an integration of social cognition, health belief, and other models and focused on eight shared variables including intention to change behavior, environmental barriers, skills, outcome expectations or attitudes, norms, self-standards, emotion, and self-efficacy. The Fishbein et al. (2001) [23] report focused on AIDS-related health behaviors that may have a similar burden of daily frequency and duration as light therapy. Neimeyer and colleagues (2008) [21] recently reviewed theoretical models appropriate for predicting homework compliance in psychotherapy and found that willingness, as well as motivation and stage of change predicted homework compliance.

### Aims of the Study

We hypothesize that the aforementioned variables such as social support, self-efficacy, and treatment credibility will predict use of LT in individuals with SAD or non-seasonal depression. Therefore, the present study sought to collect preliminary data using an anonymous web survey of individuals self-identifying as having been previously diagnosed with a disorder for which light therapy is indicated as a treatment. Specifically, we hypothesize that the decision to use LT in the previous winter would be associated with cognitive, behavioral, and social variables including self-efficacy, outcome expectations, social support, processes of change, knowledge and the degree of perceived treatment credibility. We further hypothesized that LT non-use would be associated with higher frequency of self-reported depression symptoms in the previous winter.

## Methods

### Participants

Participants were recruited through Internet websites such as online SAD-focused groups and websites for national organizations focused on support for individuals with depression. Participants completed the survey anonymously, and IP addresses were not recorded. Participants were informed that they would not be compensated for participation in order to preserve anonymity, and the survey was estimated to take about 20 minutes to complete. The study was approved and determined to be exempt from Human Subjects Research by the University of Pittsburgh Institutional Review Board, because it involved the use of survey data recorded in such a manner that human subjects cannot be identified, as no identifiers were collected. Written documentation of informed consent was waived to preserve anonymity, although information was given to participants prior to beginning the surveys regarding the duration, content, and focus of the study. Only individuals reporting a diagnosis for which LT is a treatment and those who reported having heard of LT were included in the study.

### Questionnaire Construction

Questionnaires specific to light therapy were based on existing measures described below, and were tested for psychometric properties including internal consistency as a measure of reliability and dimensionality as a measure of construct validity. Reliability was assessed with Chronbach's alpha, a measure of the intercorrelation among items in a scale, with the traditional cut off of alpha = 0.70. Scales with Chronbach's alphas that fell within the acceptable range for internal consistency (range: 0.70–0.94) were retained. Dimensionality was measured with confirmatory factor analysis, to determine if each scale is unidimensional as hypothesized, reflecting the degree of variance in the measure due to a single common factor. Principal components extraction with varimax rotation was performed, and all scales except for the knowledge scale were identified as having a single component.

With empirical evidence supporting many constructs reviewed above, some similar constructs were excluded in the present study to reduce participant burden or to address specific research hypotheses. Some measures have no clear interpretation when administered retrospectively (i.e., stage of change, intention). Others, such as attitudes, perceived behavioral control, and social norms may overlap with outcome expectations, self-efficacy, and social support scales, respectively. Therefore, the present study involved modifying a subset of all possible scales including the following existing scales to be appropriate for light therapy for depression: outcome expectations, self-efficacy, social support, knowledge, process of change, and treatment credibility. Construction methods and psychometric test results for each scale are described further below.

#### Outcome Expectations

Outcome expectations are beliefs about the efficacy and importance of a given behavior in producing desired outcomes, a construct derived from the social cognitive theory [16], [24]. This scale was modified based on one from Stepnowski et al. (2002) [17] for CPAP use in sleep apnea, and another from Gyurcsik, Brawley, Spink, Glazebrook, and Anderson (2011) [25] designed to measure outcomes related to arthritis. Three items assessed how effective participants believed regular use of light therapy is for managing mood, fatigue, and sleep, rated on a 5-point Likert scale from “not at all effective” to “extremely effective.” A fourth item assessed how important respondents believed regular use of light therapy is for managing symptoms on a 5-point scale from “not at all important” to “extremely important.” Internal consistency was acceptable (Chronbach's alpha = .84), and factor analysis revealed a single dimension for this light therapy outcome expectations scale.

#### Self-Efficacy

Self-efficacy is the belief that one can engage in a given behavior [16]. Our self-efficacy scale was modeled after those used for CPAP therapy [17] and exercise [26]. Previous items such as “I am confident I can participate in exercise when I am tired” and “I am confident I will use CPAP regularly even if I do not feel like it” were revised for light therapy (e.g., “I am confident I can use light therapy regularly even when I don't want to get up early”). Each of 5 items was scored on a 5-point Likert scale from “disagree completely” to “agree completely.” Internal consistency was high (Chronbach's alpha = .94), and factor analysis revealed a single dimension for this light therapy self-efficacy scale.

#### Social Support

Social support in this context refers to the utility of support from friends, family, and health care staff in supporting a given behavior change or adherence to a given treatment, and is also derived from the social cognitive theory [16]. The present scale was modeled on those for CPAP [17] and in light of recommendations for the assessment of social support for behavior change [16], [24]. Items such as “I have people in my life who will support me in using CPAP regularly” were revised for light therapy (e.g., “I have people in my life who support me in using light therapy regularly”). Each of the eight items was scored on a 5-point Likert scale from “disagree completely” to “agree completely.” Internal consistency was high (Chronbach's alpha = .94), and factor analysis revealed a single dimension for this light therapy social support scale.

#### Knowledge

The social cognitive theory proposes that accurate information provides the basis upon which behavior change will take place, although it is not expected to explain behavior change alone [16]. Knowledge measures the degree of accuracy of information a person has, as this may provide motivation to use a particular treatment. The first knowledge scale for this study was developed based on previously published scales and included twelve true/false questions such as “Light therapy is effective even if the user's eyes are closed” and “Normal household lights are just as effective as light boxes designed for light therapy.” This knowledge scale had a Chronbach's alpha of only. 44 and a multi-factor solution, so it was excluded from analyses. For future studies, this scale will undergo revision to reduce the level of knowledge assessed to a more basic level.

#### Processes of Change

The Transtheoretical Model combines theories of behavior change with learning theory to describe stages of motivational readiness to make a change in a given behavior [27]. Because this was a retrospective survey, the processes used to engage in change was measured, instead of the actual stage of behavioral change a given individual is in at the time of assessment. The hypothesis is that progress through the stages occurs on the basis of processes of change, or behaviors and cognitions that promote change such as reading about a particular treatment. Processes of change describe the reasons that motivate change, and the means by which it is achieved, and are associated with actual behavioral change [27]. The current processes of change questionnaire was based on previously published scales for exercise behavior [28], smoking cessation [29], and CPAP for sleep apnea [17]. Each of 20 items was rated on a 5-point Likert scale (i.e., “never,” “seldom,” “occasionally,” “often,” and “repeatedly”). Items include cognitive processes including consciousness raising, dramatic relief, environmental reevaluation, self-reevaluation, and social liberation. The scale also includes behavioral processes of change including counter-conditioning, helping relationships, reinforcement management, self-liberation, and stimulus control. Items such as this stimulus control item were revised from the original examples; “I put things around my home to remind me of exercising” [28], “I remove things from my home that remind me of smoking” [29] and “I put things around my home to remind me to use CPAP” [17], to be appropriate for light therapy (i.e., “I put my light therapy device in a place so that I'll be easily reminded to use it”). Internal consistency was high (Chronbach's alpha = .92), and factor analysis revealed a single dimension for this light therapy processes of change questionnaire.

#### The Treatment Expectations and Credibility Survey

A standardized measure of treatment credibility [30] was revised to evaluate expectations, preferences and credibility of LT as a treatment for depression. In the original Borkovec and Nau (1972) [30] measure, participants rated a new therapy intended to reduce public speaking anxiety in five questions on a 10-point Likert scale. The original included questions such as “1. How logical does this type of therapy seem to you?” and “2. How confident would you be that this treatment would be successful in eliminating fear of speaking before a group?” For the present study, we rephrased questions to reflect light therapy for depression, (e.g., 2. “How confident would you be that light therapy would be successful in eliminating depression?”). This is consistent with the treatment expectation evaluation in Michalak et al. (2007) [11] which assessed 4 items: 1) how logical the treatment seems, 2) how confident they are it would be successful, 3) how useful it might be, and 4) how confident they would be in recommending light therapy to a friend. Each of the 5 items on the scale was rated on a 10-point Likert scale from “not at all logical” to “very logical” or “not at all confident” to “very confident.” The resulting scale had acceptable internal consistency (alpha = .74) and a yielded single factor in factor analysis.

#### Depression Symptom Frequency

The Center for Epidemiologic Studies Depression Scale (CES-D) [31] is a 20 item self-report depression symptom scale developed by the National Institutes of Mental Health Center for Epidemiologic Studies. Studies have validated the CES-D as a screening tool to detect depression symptoms and to measure change in symptom severity over time [32]. This scale was not re-tested for psychometric properties in the present study, and was used instead of other measures because it does not assess suicidality, which would be difficult to respond to in an anonymous survey conducted on-line. Participants were asked to “Think about the week you felt the most depressed during the past fall-winter season. Below is a list of the ways you may have felt or behaved during that week. Please rate how often you felt the following ways during that particular week, even if you were not depressed last fall or winter.”

#### Light Therapy Usage

A series of items assessing the quantity and quality of light therapy usage by self-report were developed for the current study. A meta-analysis by Golden and colleagues (2005) [1] reported that the effective starting “dose” of light therapy ranged from 30 minutes per day to 1–2 hours, depending on the intensity of the light [1]. Participants were asked to estimate the amount of time they used light therapy in the previous winter on both weekday and weekend days, which were combined for a weighted daily average (i.e., weekday min. ×5, plus weekend min. ×2, quantity divided by 7). Because 59.5% of the sample reported not using LT in the previous winter, this variable was dichotomized into two groups: those that did use LT and those that did not use LT.

### Statistical Analyses

Multivariate analysis of variance was used to compare those that did use light therapy to those that did not use light therapy on dependent variables including total scores for processes of change, treatment credibility, outcome expectations, self-efficacy, social support, and depression symptoms, while controlling for age. The significance level was set at 0.05 for the study. Data analyses were conducted using SPSS 17.0 (SPSS Inc., Chicago, IL).

## Results

### Participants

#### Demographics

A total of 40 individuals age 18 or older responded to the survey and met study inclusion criteria. Inclusion criteria were that individuals had to self-report that they have been diagnosed with a disorder for which light therapy is a treatment (i.e., SAD or MDD), and had to report that they had at least heard of light therapy as a treatment for their disorder. Participants reported diagnoses of Major Depressive Disorder With Seasonal Pattern (MDD-SP; *n* = 16, 38.1%), Bipolar I Disorder With Seasonal Pattern (*n* = 12, 28.6%), Bipolar II Disorder With Seasonal Pattern (*n* = 9, 21.4%), or Major Depressive Disorder without a seasonal pattern (MDD; *n* = 5, 11.9%). The number of individuals with a Bipolar Disorder With Seasonal Pattern did not differ across the groups using or not using light therapy, *X^2^* (1, 42) = 0.48, *p* = .49. Similarly, the number of individuals reporting a seasonal pattern diagnosis (i.e., MDD-SP, and Bipolar I or II With Seasonal Pattern) did not differ between groups reporting use or non-use, *X^2^* (1, 42) = .26, *p* = .67. Groups that did and did not use light therapy were also compared by race, age, and gender (Table 1). As the group reporting LT use was older, age was included as a covariate in the analysis. Overall, 17 out of 40 (42.5%) respondents reported using LT the previous winter.

**Table 1:** Demographic variables compared between those that did and did not use LT.

	*Did Use LT*		*Did Not Use LT*			
Variable & Sample (*n*)	*M*	*SD*	*M*	*SD*	*F*	*p*
Age	46.65	13.24	37.24	13.06	5.19	.03*
Race	*N*	*%*	*N*	*%*	*X^2^*	*p*
White	14	82.4	19	82.6	0	1.00
Non-White	3	17.6	4	17.4		
Gender	*N*	*%*	*N*	*%*	*X^2^*	*p*
Female	15	88.2	21	91.3	.10	.75
Male	2	11.8	2	8.7		

* *p*<.05.

#### Predictors of light therapy use

**Figure 1:**
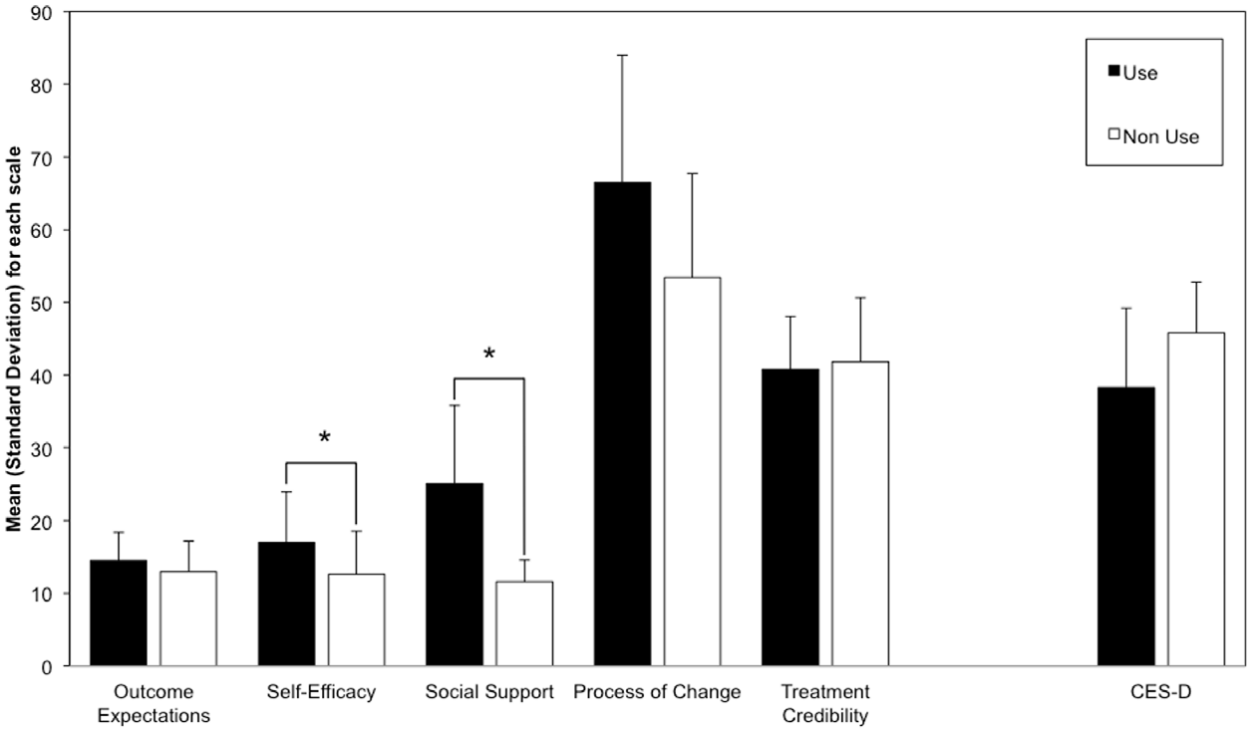
Comparison of motivation, credibility, and depression symptom scale scores between those reporting use vs. non-use of light therapy (*M, SD*). *p<.05. Each measure has a different scale and minimum/maximum values, and is only compared here between groups defined by self-reported use vs. non-use of light therapy. Use: Individuals who reported using LT. Non Use: Individuals reporting no use of LT in the previous winter.

In the omnibus MANOVA, the self-efficacy and social support scales were significantly associated with whether or not individuals reported using light therapy in the previous winter (Table 2 & Figure 1). Some other group differences were in the expected direction, albeit not significantly different across groups statistically (see Table 2).

**Table 2:** MANOVA comparing individuals that did and did not use LT.

	*Did Use LT*		*Did Not Use LT*				
Variable & Sample (*n*)	*M*	*SD*	*M*	*SD*	*F*	*p*	*η^2^*
Outcome Expectations	14.47	3.89	12.94	4.19	.01	.94	.00
Self-Efficacy	17.00	6.93	12.60	5.95	8.04	.02*	.42
Social Support	25.13	10.75	11.60	2.97	6.14	.03*	.36
Process of Change	66.50	17.49	53.40	14.36	3.70	.08	.25
CES-D	38.29	10.90	45.82	6.96	2.44	.15	.20
Treatment Credibility	40.81	7.24	41.86	8.78	.30	.60	.03

* *p*<.05.

## Discussion

Light therapy self-efficacy and social support were associated with self-report of light therapy use. These data suggest that self-efficacy and social support predict use of light therapy, just as they predict use of treatments or behavior change in other health conditions or other depression treatments. Other variables such as outcome expectations, processes of change, depression scores, and treatment credibility were not significantly different in those reporting light therapy use. Michalak et al. (2007) [11] found that treatment credibility and expectations for light therapy were not associated with adherence to light therapy in SAD, consistent with our findings. In that study, as well as ours, scores on the treatment expectations scale were high in relation to the maximum score possible on each scale. In our study, the group mean scores on treatment credibility approached the maximum possible score of 10 across all 5 items (Did use: *M* = 8.16, *SD* = 1.45; Didn't use: *M* = 8.37, *SD* = 1.76). Therefore, it appears that treatment credibility is high across both samples, and a ceiling effect may explain the lack of predicted association between treatment credibility and LT use.

The main limitation is that the sample used was a self-selected group of individuals that self-identified as having been diagnosed with a mood disorder for which LT is recommended, rather than a group of clinically diagnosed participants. However, means and standard deviations for the CES-D scores were 38.29 (10.90) for the group reporting light therapy use, and 45.82 (6.96) for the group reporting no use, indicating that individuals were reporting significant depression symptom frequency for the previous winter. Individuals in this study reported similar symptom levels to those with a diagnosed mood disorder (*M* = 38.1 for individuals in a mood episode) [32]. Because self-reported diagnosis may be inaccurate, future studies will include in-person structured clinical interviews for diagnosis.

Another limitation of this study is that only 42.5% of the respondents reported using light therapy, so our measures are predicting any amount of light therapy use, rather than degree of adherence to a light therapy prescription. Additionally, the present study did not measure factors associated with adherence to other medication or psychotherapy treatments for depression, such as intention and willingness. These constructs may overlap with those of process of change, treatment credibility, and outcome expectations. Intention and willingness are related but theoretically separate constructs of behavioral change that have been defined as follows. Willingness reflects how willing an individual would be to engage in a particular coping strategy if a friend or treatment provider suggested it [33]. On the other hand, the construct of intention reflects a person's intention to perform a specific behavior (e.g., “I intend to do X”) [34]. Multiple theories including cognitive attitude-behavior relations, models of health behavior, and goal theory all propose that one's intention to complete homework determines motivation and performance [see 35–38]. In a meta-analysis of studies measuring intention and behavior change, intention accounted for 28% of the variance in behavior [39]. Burns and Nolen-Hoeksema (1991) [40] found that willingness and homework compliance individually predicted clinical improvement in CBT for depression. Because the present study is retrospective, and interpretation of intent retrospectively would be difficult to interpret, we measured overall willingness to engage in coping strategies for depressed mood. Future studies could measure intention and willingness at the time of diagnosis of a mood disorder, before a trial of light therapy, if patients who had not previously been diagnosed could be recruited.

In some ways, it is surprising that individuals choose not to use light therapy given that side effects are generally mild [3], [41], and that the rationale linking seasonal recurrence to treatment with light seems credible [42]. However, we found that other factors besides low side effects and treatment credibility that may also be important, namely self-efficacy and social support. Therefore, interventions that manipulate these motivational, cognitive, and behavioral factors may increase LT use rates, such as Motivational Enhancement Therapy [43] or Motivational Interviewing [44]. These approaches have demonstrated efficacy for a variety of health and mental health conditions for which successful treatment requires complex and sustained behavior change [45]. Understanding the impact of thoughts and appraisals regarding symptoms and treatment on the decision to use LT could be used to inform cognitive-behavioral interventions to maximize LT use and improve treatment in mood disorders. Further measurement of cognitive behavioral predictors of LT use should be performed prospectively with participants who have undergone structured diagnostic interviews, to increase confidence in the relationships between self-efficacy and social support and the use of LT.
